# Supplementary material for: Comparative Evaluation of Recombinant and Acellular Pertussis Vaccines in a Murine Model
Source: Vaccines (Basel). 2024 Jan 22;12(1):108. doi: 10.3390/vaccines12010108 (PMC10818713; doi:10.3390/vaccines12010108)
Supplement: Supplementary file 1 [file vaccines-12-00108-s001.zip › vaccines-2756284-supplementary.pdf]

a

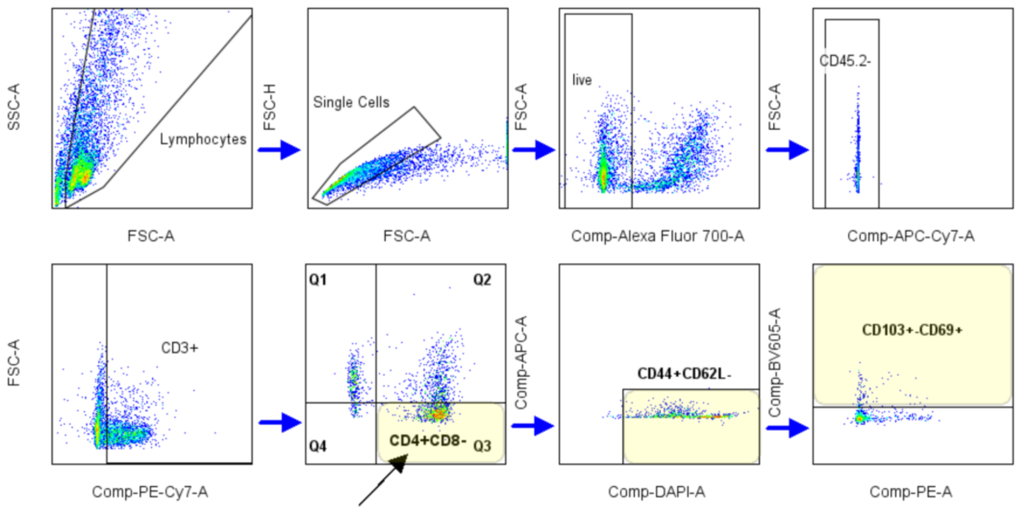

b

Fluorochrome	Marker
APC-R700	Alexa Fluor 700-A
APC-Cy7	CD45.2
FITC	CD4
PE-Cy7	CD3
BB700 / PE Cy5	CD8
BV421 / DAPI	CD44
BV605	CD69
PE	CD103
APC	CD62L

Figure S1 : Flow cytometry gating strategy for TRM CD4+ T cells. CD4+ T cells and T RM cells in lungs from 5 mouse per each group were analyzed with FACS Aria Fusion at 3 time points. The cells were analyzed with FACS Aria Fusion. 10 minutes before sacrifice all mice were injected CD45.2 at tail vein. **(a)** Flow cytometry gating strategy for TRM CD4+ T cells. The lymphocytes were gated widely by forward (FSC)-A and side (SSC)-A. Lymphocytes were plotted against a FCS-A vs FSC-H for single cells. A live/dead cell gate was applied based on Alexa fluor 700 (APC R700). Impermeable to APC R700 presents live cells. The live cells were plotted against CD45.2 vs FSC-A gate for lung tissue retained cells. Lung tissue retained cells were plotted against CD3 vs FSC-A for CD3+ T cells gating. After, sorting CD3+ T cells, CD4 vs CD8 gate was applied. The CD4+ T cells were assessed by CD44 and CD62L expressions which enabled the central memory T cell (TCM) (CD44+CD62L+) and effector memory T cell (TEM)(CD44+CD62L-) cells. Among the TEM cells, TRM CD4+ T cells were gated with CD103 and CD69. CD103+ CD69+ cells and CD103-CD69+ cells were sorted as T RM cells. (**b**) Fluorochrome and marker used in the TRM CD4+ T cells assay.

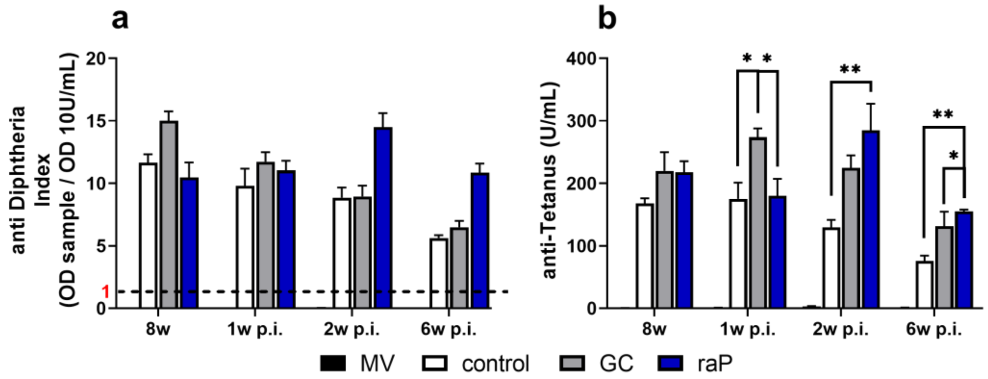

Figure S2. : Diphtheria and Tetanus Humoral responses. Serum was collected at 8 weeks and at 1, 2, and 6 weeks post infection (p.i.) with pertussis ATCC 9797, and the antibody titers against diphtheria and tetanus were analyzed. Five animals were used per group, and each sample was diluted in a 1:20,000 ratio. (a) For the diphtheria results, the sample OD was divided by the 10 U/mL OD to calculate the index, and a result of 1 or greater is interpreted as positive. (b) For tetanus, a calibrator curve was plotted by point-to-point interpolation, and the results were derived in U/mL compared to the calibrator curve. Between-group differences were investigated using one-way ANOVA and Bonferroni's post hoc test, and P values have been displayed as follows: $*p < 0.05$, $**p < 0.01$. Differences with the saline group were excluded.

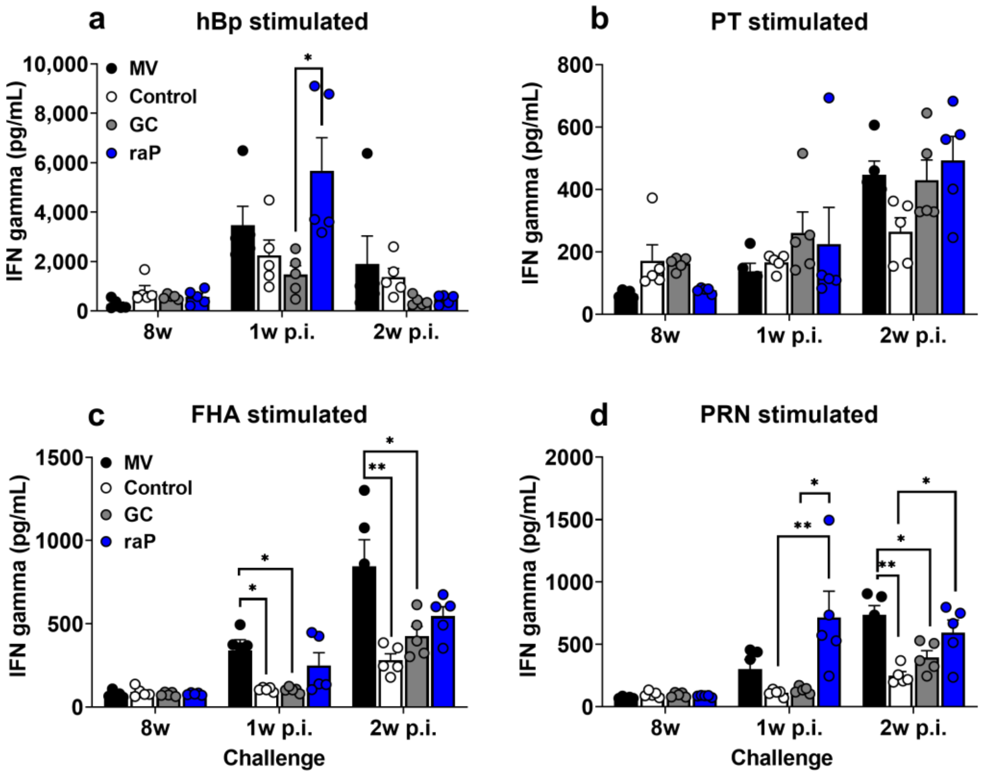

Figure S3. IFN-gamma cytokine secretion. Spleen samples were collected at 3 weeks after the first and second injections with the primary vaccine and the booster vaccine (8 weeks), and at 1 and 2 weeks post-infection with pertussis (ATCC 9797). Supernatant from the samples was collected, and IFN-gamma cytokine secretion was analyzed using an ELISA kit (Proteintech). Five animals were used in each group. (a) heat inactivated Bordetella pertussis (b) Pertussis toxoid (c) FHA (d) PRN stimulated. Between-group differences were investigated using one-way ANOVA and Bonferroni's post hoc test, and P

values have been displayed as follows: *$p < 0.05$, **$p < 0.01$. Differences with the saline group have been omitted.

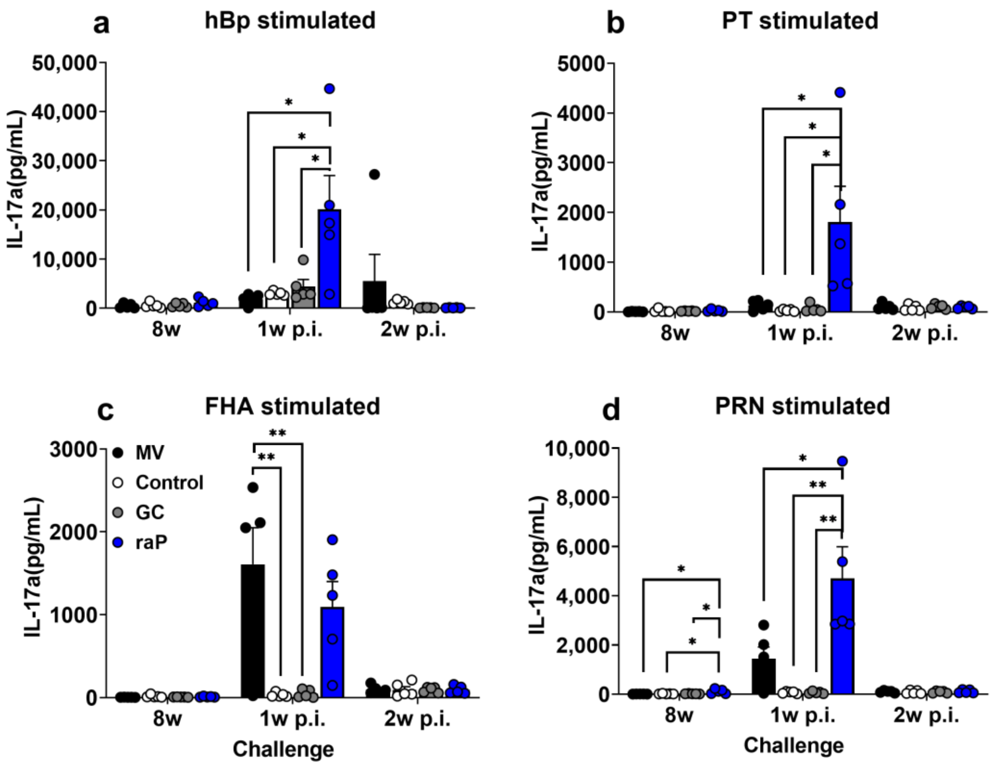

Figure S4. IL-17a cytokine secretion. Spleen samples were collected at 3 weeks after the first and second injections with the primary vaccine and the booster vaccine (8 weeks), and at 1 and 2 weeks post-infection with pertussis (ATCC 9797). Supernatant from the samples was collected, and IL-17a cytokine secretion was analyzed using an ELISA kit (Proteintech). Five animals were used in each group. (a) heat inactivated Bordetella pertussis (b) Pertussis toxoid (c) FHA (d) PRN stimulated. Between-group differences were investigated using one-way ANOVA and Bonferroni's post hoc test, and P values have been displayed as follows: *$p < 0.05$, **$p < 0.01$. Differences with the saline group have been omitted.
